# Technology addiction as a predictor of visual problems in early adolescents: a multicentre case–control study

**DOI:** 10.1017/S1463423626101546

**Published:** 2026-07-28

**Authors:** Ecem Çiçek, Feyza Demir Bozkurt, Hilal Büyüktopaç Çakıcı

**Affiliations:** Department of Nursing, Faculty of Health Science, Bartın University, Türkiye

**Keywords:** early adolescents, primary health care, public health nursing, school nursing, screen time, technology addiction, visual health

## Abstract

Increasing screen exposure among early adolescents has raised concerns regarding technology addiction and its potential impact on visual health. Within the scope of primary health care, early identification of modifiable behavioural risk factors is essential for preventive intervention. This case–control study examined the association between technology addiction and visual problems among early adolescents and explored implications for primary health care and school health services. The study included 585 students in grades 5–8 from three randomly selected public middle schools. The case group comprised 47 students diagnosed with visual impairment or wearing corrective lenses, while the control group included 538 students without visual problems. Data were collected via face-to-face interviews using a Personal Information Form and the Technology Addiction Scale. Logistic regression analysis was conducted to determine predictors of visual problems. Students with visual problems demonstrated significantly higher total technology addiction scores and higher subdimension scores for social media use, instant messaging, online gaming, and website browsing (*p* < .001). Longer daily screen time and irregular sleep patterns were associated with increased addiction levels. Gender differences were observed: girls scored higher in instant messaging and website use, whereas boys scored higher in gaming addiction. Online gaming duration and total addiction scores were significant predictors of visual problems. Technology addiction, particularly excessive gaming exposure, appears to be a modifiable risk factor for visual problems in early adolescence. Integrating digital hygiene education, screen-time monitoring, and behavioural risk assessment into primary health care and school nursing practice may contribute to early prevention and health promotion strategies.

## Introduction

The rapid advancement of information and communication technologies has profoundly influenced individuals’ behaviours and habits across all areas of life (OECD, 2021). Today, technology has become an indispensable component of many domains, including education, healthcare, work life, and social interactions (Hooft Graafland, 2018). In line with this transformation, a notable increase in internet usage has been observed among individuals of all ages worldwide, including in Türkiye (TUİK, 2023). Early adolescence is a critical developmental stage during which biological, psychological, and social changes occur rapidly. During this period, adolescents’ engagement with technology intensifies, with mobile devices, social media, and video games playing a significant role in their daily lives (Valkenburg and Peter, 2013). Consequently, for children and adolescents who have been born into and are growing up in a digital world, technology increasingly permeates their lives, both positively and negatively (Reid Chassiakos *et al*., 2016; Livingstone and Blum-Ross, 2020).

The central role of technology in daily life has introduced several risks, particularly for children and adolescents. Excessive and purposeless use of technology may foster addictive behaviours, contributing to increased screen time and technology dependency among young individuals (Reid Chassiakos *et al.*, 2016). The World Health Organization (WHO, 2019) defines screen time as the time spent passively watching screen-based entertainment on devices such as televisions, computers, or mobile phones, explicitly excluding physically active screen-based games from this definition. In a study conducted among middle school students in Türkiye, 10.7% were identified as internet-dependent, and 11.7% were considered at risk (Ulaş Karaahmetoğlu, 2021). A growing body of research has reported that technology addiction may have direct and indirect negative effects on the physical and psychological health of children and adolescents (Paulich *et al*., 2021; Priftis and Panagiotakos, 2023). Increased screen time and digital device dependency have been associated with various vision-related problems, such as digital eye strain (Reid Chassiakos *et al.*, 2016) and myopia, and may adversely impact developmental outcomes, academic performance, and overall quality of life among youth (Paulich et al., 2021; Jain *et al*., 2023; Priftis and Panagiotakos, 2023).

Recent studies have shown that the increase in screen time, along with rising digital addiction, contributes to the growing prevalence of visual problems such as digital eye strain and myopia among children (Foreman *et al*., 2021). For example, a comprehensive meta-analysis conducted by Ha *et al*. (2025) revealed that each additional hour of daily screen time increases the risk of myopia in children by 21%. In particular, studies conducted in regions with high rates of internet and gaming addiction – such as Southeast Asia – have reported that 19% of students experience symptoms of DES (Balhara *et al*., 2018). Digital eye strain is defined as a clinical condition resulting from prolonged use of digital screens and includes symptoms such as blurred vision, dry eyes, headaches, and difficulty focusing (Almahmoud *et al*., 2025).

The relationship between technology addiction and visual problems in early adolescents remains insufficiently explored. Understanding the link between vision health and technology use is crucial for protecting well-being during this critical developmental period. Furthermore, early interventions must be developed to prevent and manage such health issues (Oswald *et al*., 2020). In this context, school nurses play a vital role in monitoring students’ health and providing early interventions. They contribute to preventive nursing practices by conducting vision screenings and delivering health education aimed at increasing students’ awareness of technology use (NASN, 2012; Murphy, 2016). While numerous studies have investigated internet, technology, or gaming addiction and their associated physical and psychosocial factors in adolescents (George *et al*., 2018; Ulaş Karaahmetoğlu, 2021; Jain *et al.*, 2023), few have specifically examined the impact of technology addiction levels on visual problems. Therefore, the present study aimed to evaluate the relationship between technology addiction levels and visual problems among early adolescents in middle schools using a multicentre school-based case–control design.

## Method

### Study design

This study was designed as a multicentre school-based case–control study conducted among early adolescents attending public middle schools. The term retrospective was removed from the manuscript to avoid confusion, as data were collected through school-based vision screening and face-to-face surveys during the study period. This study was defined as retrospective because the case and control groups were formed based on existing school vision screening records and survey data collected during the 2022–2023 academic year. No prospective follow-up or intervention was conducted. Instead, previously collected screening and questionnaire data were reviewed retrospectively to examine whether technology addiction was associated with visual problems among early adolescents. In addition, the study planed as retrospective, because its main purpose was to compare students with and without visual problems and to examine whether technology addiction-related factors were associated with case group membership. Thus, a retrospective case–control design was considered the most appropriate approach for the research aim.

### Participants

The study population consisted of students enrolled in the 5th, 6th, 7th, and 8th grades of public middle schools located in the central district of Bartın Province during the 2022–2023 academic year. Three public middle schools were selected using a random sampling method. Since the study was conducted within the scope of school-based vision screening and aimed to include all eligible students in the selected schools, an a priori sample size calculation was not performed. Instead, a total population sampling approach was used. All students who met the inclusion criteria, volunteered to participate, and had written parental consent were included in the study. The final sample consisted of 585 students.

The case group included students who were identified as having visual problems during school-based visual acuity screening or who reported wearing corrective lenses, whereas the control group included students without visual problems. Visual problems were determined using predefined school-based screening criteria rather than comprehensive ophthalmologic diagnosis. Students with visual acuity of 20/30 (6/10) or better in both eyes, with no more than a two-line difference between the eyes, were classified as having normal vision. Students with visual acuity of 20/30 (6/10) or worse in either eye, or a difference of two or more lines between the two eyes, were classified as having visual problems.

The inclusion criteria were as follows: (a) being a student at one of the selected schools, (b) having written parental consent, (c) volunteering to participate, (d) having no speech communication impairment, and (e) having no intellectual disability.

### Research questions

1. To what extent is technology addiction observed among early adolescents attending secondary school?
2. What is the prevalence of visual problems among early adolescents?
3. Is there a relationship between technology addiction and visual problems in early adolescents?
4. Which factors significantly predict the likelihood of visual problems among early adolescents?

#### Data collection tools

1. **Survey form:** A 16-item survey form was created based on the literature to assess the demographic characteristics of participants.
2. **Technology addiction scale:** The Technology Addiction Scale, developed by Aydın (2017), measures the level of technology addiction with four subscales: Social Network Addiction, Instant Messaging Addiction, Online Game Addiction, and Website Addiction. The scale consists of 24 items and uses a 5-point Likert scale. The scale interpretation is as follows: 0–24: ‘Not addicted’ 25–48: ‘Low level of addiction’ 49–72: ‘Moderate level of addiction’ 73–96: ‘Quite addicted’ 97–120: ‘Fully addicted’. While the overall Cronbach’s alpha value of the scale was previously reported as .79, it was calculated as .895 in this study, indicating a high level of internal consistency.

*Implementation of the study*: Due to the absence of school nurses or health units in the Implementation of the Study: Due to the absence of school nurses or health units in the participating schools, preliminary meetings were organized with school administrators and teachers to explain the eye screening procedures. Screening schedules were arranged at convenient times. Prior to data collection, a quiet, well-lit, and safe physical environment was established. Eye screenings were conducted in small groups under the supervision of classroom teachers. The researchers trained student assistants responsible for data collection in the correct use of eye charts and the standardized procedures for conducting the screenings.

Students identified as having potential visual problems through screening or corrective lens use were included in the case group for research purposes. Students with normal visual acuity according to the predefined screening criteria were included in the control group. Because the classification was based on school screening rather than a full ophthalmologic examination, the term visual problems was used to describe screening-based visual impairment risk. Informational and consent forms were distributed to the families of students in both groups, and the survey form was completed by the students.

*Data analysis*: The data were analysed using Statistical Package for the Social Sciences (SPSS) version 26.0. Descriptive statistics such as frequencies, percentages, means, and standard deviations were reported. The normality of the data was tested, and group comparisons were made using Student’s *t*-test, ANOVA, correlation analysis, and binary logistic regression. For comparisons of nominal or ordinal data between groups, Pearson’s chi-square test and Fisher’s exact test were used. Odds ratios with 95% confidence intervals were reported for the logistic regression model. To correct for the imbalance between the case and control groups, sample weights were applied as described by Zhou *et al*. (2022). To account for the imbalance between the case group (*n* = 47) and the control group (*n* = 540), weighted case analysis was applied in all relevant statistical procedures. A 4:1 control-to-case weighting ratio was used, in line with standard epidemiological comparison practices. The total control group weight was adjusted to four times the number of cases (4 x 47 = 188), and the individual weight for control group participants was calculated as 188/540 ≈ 0.348. In SPSS, a custom weight variable was computed using the formula: weight = (grou*p* = 1) * 1 + (grou*p* = 2) * 0.348. This weighting ensured that both groups contributed proportionally to the analyses, without inflating the sample size. All inferential analyses-including independent-samples *t*-tests, one-way ANOVA, and regression models-were conducted on the weighted dataset using SPSS’s ‘Weight Cases’ procedure. Descriptive statistics are reported as unweighted data.

*Ethical considerations and institutional approval:* The study was approved by the Ethics Committee of the Social and Human Sciences at XXX University, with approval number 2022-SBB-0661, dated 30.12.2022. Written consent was obtained from the participating schools, and parental consent was secured for all student participants. The confidentiality of the participants was maintained, and their personal information was used exclusively for the purposes of the research. The study adhered to the principles of the Declaration of Helsinki and national ethical standards.

## Results

The average age of the students participating in the study was determined to be 11.81 years. 47.5% of the participants were female students. When examining the overall level of technology addiction, it was found that 56.2% of the students had low levels of addiction, while 33.4% had moderate levels of addiction. According to the analysis, significant differences were found in the levels of instant messaging, online game, and website addiction based on the gender of the participants (Table 1).

**Table 1.** Distribution of case-control groups according to demographic characteristics Table 1 long description.

	Overall (*n* = 587)	Control (*n* = 540)	Case (*n* = 47)	
Characteristics	*n* (%)	*n* (%)	*n* (%)	F, X ^2^ /*p*-Value
**Gender**				
Girl	279 (47.5)	253 (43.1)	26 (4.4)	1.243/.168
Boy	308 (52.5)	287 (48.9)	21 (3.6)	
**Classroom**				
5	224 (38.2)	214 (36.5)	10 (1.7)	**3.072/.027**
6	166 (28.3)	152 (25.9)	14 (2.4)	
7	144 (24.5)	125 (21.3)	19 (3.2)	
8	53 (9.0)	49 (8.3)	4 (0.7)	
**Mother’s education level**				
primary school graduate	148 (25.3)	136 (23.2)	12 (2.1)	.405/.805
secondary school graduate	122 (20.8)	11 (1.9)	111 (18.9)	
high school graduate	192 (32.7)	12 (2.0)	180 (30.7)	
university graduate	125 (21.3)	12 (2.0)	113 (19.3)	
**Father’s education level**				
primary school graduate	86 (14.6)	78 (13.3)	8 (1.4)	.174/.952
secondary school graduate	111 (18.9)	104 (17.7)	7 (1.2)	
high school graduate	221 (37.6)	203 (34.6)	18 (3.1)	
university graduate	169 (28.8)	155 (26.4)	14 (2.4)	
**Income status**				
Income less than expenditure	39 (6.6)	38 (6.5)	1 (0.2)	2.545/.079
Income equal to expenditure	350 (59.6)	315 (53.7)	35 (6.0)	
Income more than expenditure	198 (33.7)	187 (31.9)	11 (1.9)	
**Owning a smartphone**				
Yes	456 (77.7)	417 (71.0)	39 (6.6)	.837/.236
No	131 (22.3)	123 (21.0)	8 (1.4)	
**Owning a desktop computer**				
Yes	157 (26.7)	145 (24.7)	12 (2.0)	.038/.499
No	430 (73.3)	395 (67.3)	35 (6.0)	
**Owning a laptop**				
Yes	264 (45.0)	237 (40.4)	27 (4.6)	3.212/.051
No	323 (55)	303 (51.6)	20 (3.4)	
**Average time spent on tablet computer**				
Less than 1 hour	381 (64.9)	362 (61.7)	25 (4.3)	3.594/.007*
1–2 hours	141 (24.0)	116 (19.8)	11 (1.9)	
3–4 hours	38 (6.5)	40 (6.8)	7 (1.2)	
5–6 hours	12 (2.0)	17 (2.9)	1 (0.2)	
7 hours or more	15 (2.6)	5 (0.9)	3 (0.5)	
**Average time spent on laptop**				
Less than 1 hour	381 (64.5)	360 (61.3)	21 (3.6)	4.909/.001**
1-2 hours	141 (24.0)	126 (21.5)	15 (2.6)	
3-4 hours	38 (6.5)	34 (5.8)	4 (0.7)	
5-6 hours	12 (2.0)	10 (1.7)	2 (0.3)	
7 hours or more	15 (2.6)	10 (1.7)	5 (0.9)	
**Average time spent on smartphone**				
Less than 1 hour	140 (23.9)	126 (21.5)	14 (2.4)	3.241/.012*
1-2 hours	208 (35.4)	199 (33.9)	9 (1.5)	
3-4 hours	160 (27.3)	149 (25.4)	11 (1.9)	
5-6 hours	52 (8.7)	42 (7.2)	9 (1.5)	
7 hours or more	28 (4.8)	24 (4.1)	4 (0.7)	
**Average time spent in online games**				
Less than 1 hour	286 (48.7)	273 (46.5)	13 (2.2)	5.019/.001**
1-2 hours	182 (31.0)	168 (28.6)	14 (2.4)	
3-4 hours	69 (11.8)	58 (9.9)	11 (1.9)	
5-6 hours	30 (5.1)	26 (4.4)	4 (0.7)	
7 hours or more	20 (3.4)	15 (2.6)	5 (0.9)	
**Daily sleep duration (%)**				
Less than 8 hours	113 (19.3)	225 (17.9)	8 (1.4)	.088/.916
8-10 hours	404 (68.8)	371 (63.2)	33 (5.6)	
More than 10 hours	70 (11.9)	64 (11.9)	6 (1.0)	
**Recent occurrence of eye pain or itching**				
Yes	73 (12.4)	67 (11.4)	6 (1.0)	.840/.432
Rarely	214 (36.5)	193 (32.9)	21 (3.6)	
No	300 (51.1)	280 (47.7)	20 (3.4)	
**Prevalence of technology addiction**				
Not Addicted	9 (1.5)	8 (1.5)	1 (2.1)	5.478/.000**
Low Level Addiction	330 (56.2)	314 (58.1)	16 (34.0)	
Moderate Level Addiction	196 (33.4)	175 (32.4)	21 (44.7)	
Highly Addicted	42 (7.2)	37 (6.9)	5 (10.6)	
Fully Addicted	10 (1.7)	6 (1.1)	4 (8.5)	
Age (mean ± SD*)	11.81 ± 1.05	11.78 ± 1.05	12.19 ± 0.90	

SD: Standard deviation; *One-Way ANOVA, **Pearson Chi-square, *p < .05, **p < .001.

The analysis of the Technology Addiction Scale scores according to various demographic variables is presented in Table 2. Significant differences were observed between case and control groups across all subdimensions of technology addiction. The case group had statistically higher mean scores in social media addiction (M = 15.0, SD = 4.9), instant messaging addiction (M = 14.8, SD = 5.3), online gaming addiction (M = 14.5, SD = 6.3), website addiction (M = 13.8, SD = 6.6), and total technology addiction (M = 58.1, SD = 19.8) compared to the control group (*p* < .05). In terms of gender, boys had significantly higher scores than girls in online gaming addiction (*p* < .001), while no significant gender-based differences were found in other subdimensions. When examining educational stages, only online gaming addiction scores showed a statistically significant difference (*p* = .031), with older students tending to report higher addiction levels. No statistically significant differences were found in technology addiction scores based on smartphone, desktop, or laptop ownership (*p* > .05). However, higher screen time was consistently associated with increased technology addiction scores. For instance, students who used desktop computers, smartphones, laptops, or engaged in online gaming for more than 5 hours per day had significantly higher mean scores in all addiction subdimensions and total scores (*p* < .01) (Table 2).

**Table 2.** Analysis of demographic variables related to the technology addiction scale (*n* = 235) Table 2 long description.

Variables	Technology addiction scale					
		Social media addiction	Instant messaging addiction	Online gaming addiction	Website addiction	Total
	*n (%)	(Mean ± SD)	(Mean ± SD)	(Mean ± SD)	(Mean ± SD)	(Mean ± SD)
**Group**						
**Case**	47 (20)	15.0 ± 4.9	14.8 ± 5.3	14.5± 6.3	13.8 ± 6.6	58.1 ± 19.8
**Control**	188 (80)	12.3 ± 4.8	12.3 ± 5.0	11.7 ± 5.6	11.3 ± 5.1	47.5 ± 16.5
**t/p		3.524/0.001**	3.034/0.003**	2.996/0.003**	2.449/0.017*	3.777/0.000**
**Gender**						
Girl	114 (48.5)	13.0 ± 5.1	13.3 ± 5.6	10.7 ± 5.1	12.3 ± 6.0	49.3 ± 18.8
Boy	121 (51.5)	12.6 ± 4.8	12.2 ± 4.7	13.7 ± 6.1	11.3 ± 5.0	49.8 ± 16.7
**t/p		0.604/0.546	1.571/0.118	4.099/0.000**	1.480/0.140	0.214/0.831
**School**						
**1**	23 (9.6)	10.98 ± 4.08	11.22 ± 4.88	9.25 ± 4.59	10.92 ± 5.19	42.37 ± 16.46
**2**	126 (53.7)	12.98 ± 5.05	12.67 ± 5.26	12.74 ± 6.00	11.82 ± 5.48	50.22 ± 18.05
**3**	86 (36.7)	13.05 ± 4.87	13.29 ± 5.06	12.27 ± 5.66	11.97 ± 5.70	50.58 ± 17.16
***F/p		1.744/0.177	1.492/0.227	3.527/0.031*	0.324/0.723	2.115/0.123
				**1 < 2**		
**Owning a smartphone**						
Yes	184 (78.2)	12.98 ± 4.98	13.02 ± 5.24	12.32 ± 5.95	12.01 ± 5.67	50.32 ± 18.21
No	51 (21.6)	12.26 ± 4.66	11.76 ± 4.77	11.97 ± 5.36	10.96 ± 4.92	46.95 ± 15.49
**t/p		0.929/0.354	1.535/0.126	0.373/0.709	1.202/0.231	1.204/0.230
**Owning a desktop computer**						
Yes	62 (26.6)	13.26 ± 4.72	13.25 ± 5.05	12.82 ± 6.27	12.26 ± 5.86	51.58 ± 18.35
No	172 (73.4)	12.65 ± 4.99	12.58 ± 5.21	12.02 ± 5.65	11.62 ± 5.40	48.88 ± 17.41
**t/p		0.838/0.403	0.876/0.382	0.925/0.356	0.776/0.439	1.037/0.301
**Owning a laptop**						
Yes	109 (46.6)	12.92 ± 5.16	12.78 ± 5.21	12.29 ± 6.27	11.90 ± 5.66	49.89 ± 18.98
No	125 (53.4)	12.73 ± 4.71	12.73 ± 5.14	12.18 ± 5.42	11.70 ± 5.41	49.34 ± 16.52
**t/p		0.295/0.768	0.072/0.942	0.145/0.885	0.281/0.779	0.239/0.812
**Income status**						
Income less than expenditure	14 (6.1)	13.05 ± 4.96	12.82 ± 5.19	11.56 ± 5.60	11.72 ± 5.81	49.15 ± 17.83
Income equal to expenditure	145 (61.6)	13.21 ± 4.84	13.26 ± 4.98	12.79 ± 5.95	12.24 ± 5.52	51.50 ± 17.35
Income more than expenditure	76 (32.4)	12.03 ± 5.02	11.79 ± 5.43	11.30 ± 5.54	10.94 ± 5.45	46.05 ± 17.93
***F/p		1.447/0.237	2.044/0.132	1.748/0.176	1.383/0.253	2.392/0.094
**Mother’s education level**						
primary school graduate	59 (25.3)	13.36 ± 4.88	13.23 ± 4.60	12.73 ± 5.59	12.56 ± 6.13	51.03 ± 16.47
secondary school graduate	50 (21.1)	13.43 ± 4.56	12.85 ± 4.39	11.58 ± 5.10	11.30 ± 5.21	49.16 ± 14.63
high school graduate	75 (31.8)	12.85 ± 4.88	13.37 ± 5.82	12.54 ± 5.82	12.33 ± 5.86	51.08 ± 18.96
university graduate	51 (21.8)	12.22 ± 5.19	11.95 ± 5.36	12.64 ± 6.81	11.69 ± 5.76	48.50 ± 19.97
***F/p		0.933/0.446	0.698/0.594	0.490/0.743	0.615/0.652	0.604/0.660
**Father’s education level**						
primary school graduate	35 (14.9)	14.30 ± 0.09	14.84 ± 0.51	14.21 ± 0.67	14.83 ± 0.51	58.18 ± 0.66
secondary school graduate	43 (18.4)	12.71 ± 4.76	12.77 ± 5.21	12.07 ± 5.40	11.56 ± 5.67	49.11 ± 17.34
high school graduate	89 (37.7)	12.64 ± 4.58	12.63 ± 4.83	11.55 ± 5.44	11.48 ± 5.30	48.30 ± 16.70
university graduate	68 (28.9)	12.46 ± 5.23	12.49 ± 5.63	12.91 ± 6.69	11.67 ± 5.72	49.53 ± 19.65
***F/p		0.696/0.596	0.598/0.665	0.938/0.443	1.122/0.347	0.939/0.442
**Average time spent on desktop computer**						
Less than 1 hour (a)	151 (64.3)	12.32 ± 4.89	12.26 ± 5.10	11.40 ± 5.56	11.12 ± 5.45	47.10 ± 17.41
1–2 hours (b)	51 (21.9)	12.41 ± 4.45	13.04 ± 5.04	12.52 ± 5.54	12.28 ± 5.54	50.24 ± 16.91
3–4 hours (c)	21 (8.8)	15.55 ± 4.45	14.08 ± 4.76	14.09 ± 5.01	13.28 ± 5.07	57.00 ± 13.67
5–6 hours (d)	7 (2.9)	15.64 ± 5.41	15.44 ± 6.09	17.44 ± 7.48	13.55 ± 5.96	62.07 ± 17.69
7 hours or more (e)	5 (2.0)	17.22 ± 6.60	15.68 ± 7.77	19.62 ± 8.58	18.28 ± 3.73	70.81 ± 24.69
***F/p		3.744/0.006**	1.587/0.179	4.929/0.001**	2.927/0.022*	4.487/0.002**
		**(a) < (c)**		**(a) < (d), (a) < (e)**	**(a) < (e)**	**(a) < (e)**
**Average time spent on smartphone**						
Less than 1 hour	58 (24.6)	11.96 ± 4.55	11.28 ± 4.59	11.38 ± 4.80	10.36 ± 4.79	44.99 ± 14.39
1–2 hours	78 (33.3)	12.00 ± 4.60	12.31 ± 4.76	10.99 ± 5.22	10.62 ± 4.74	45.92 ± 16.10
3–4 hours	63 (26.8)	12.50 ± 4.28	12.56 ± 4.84	12.12 ± 5.89	12.14 ± 5.32	49.33 ± 15.61
5–6 hours	24 (10.1)	15.54 ± 5.57	16.09 ± 6.10	15.54 ± 6.43	16.09 ± 6.92	63.26 ± 21.21
7 hours or more	12 (5.3)	18.39 ± 5.51	17.11 ± 5.64	18.27 ± 6.98	15.89 ± 5.77	69.66 ± 19.49
***F/p		7.570/0.000**	6.539/0.000**	7.082/0.000**	8.050/0.000**	10.898/0.000**
		**(a) < (d), (a) < (e), (b) < (d), (b) < (e), (c) < (e)**	**(a) < (d), (a) < (e), (b) < (d), (b) < (e), (c) < (d), (c) < (e)**	**(a) < (d), (a) < (e), (b) < (d), (b) < (e)**	**(a) < (d), (a) < (e), (b) < (d), (b) < (e), (c) < (d)**	**(a) < (d), (a) < (e), (b) < (d), (b) < (e), (c) < (d), (c) < (e)**
**Average time spent on laptop**						
Less than 1 hour (a)	146 (62.3)	12.40 ± 4.64	12.00 ± 4.77	11.36 ± 5.26	11.18 ± 5.21	46.95 ± 16.02
1–2 hours (b)	59 (25.1)	12.53 ± 4.87	13.55 ± 4.98	11.67 ± 5.11	11.78 ± 5.34	49.52 ± 16.90
3–4 hours (c)	16 (6.7)	14.31 ± 5.64	14.30 ± 7.25	14.74 ± 6.31	12.94 ± 6.01	56.29 ± 20.94
5–6 hours (d)	5 (2.3)	12.98 ± 3.96	12.48 ± 3.52	16.75 ± 5.01	12.58 ± 3.75	54.79 ± 9.68
7 hours or more (e)	8 (3.6)	19.02 ± 5.35	17.58 ± 6.46	23.54 ± 5.94	19.76 ± 6.50	79.91 ± 18.74
***F/p		4.270/.002**	3.477/.009*	12.550/.000**	5.425/.000**	8.741/.000**
		**(a) < (e), (b) < (e)**	**(a) < (e)**	**(a) < (e), (b) < (e), (c) < (e)**	**(a) < (e), (b) < (e), (c) < (e)**	**(a) < (e), (b) < (e), (c) < (e), (d) < (e)**
**Average time spent in online games**						
Less than 1 hour	108 (46.0)	11.42 ± 4.75	11.71 ± 5.16	9.79 ± 4.87	10.75 ± 5.42	43.66 ± 17.08
1–2 hours	72 (30.8)	12.90 ± 4.36	13.13 ± 4.88	12.23 ± 4.70	11.57 ± 4.48	49.84 ± 13.52
3–4 hours	31 (13.3)	14.64 ± 4.35	13.63 ± 4.83	15.69 ± 4.62	12.76 ± 6.06	56.72 ± 15.71
5–6 hours	13 (5.6)	15.97 ± 5.59	14.31 ± 4.63	17.64 ± 7.46	14.40 ± 6.48	62.31 ± 19.92
7 hours or more	10 (4.4)	17.33 ± 5.44	16.53 ± 6.61	20.60 ± 6.44	18.11 ± 5.59	72.57 ± 19.89
***F/p		7.485/0.000**	3.185/0.014*	20.833/0.000**	5.724/0.000**	12.270/0.000**
		**(a) < (b), (a) < (c), (a) < (d), (b) < (d),**	**(a) < (e)**	**(a) < (b), (a) < (c), (a) < (d), (a) < (e), (b) < (c), (b) < (e), (c) < (e)**	**(a) < (e), (b) < (e), (c) < (e), (d) < (e)**	**(a) < (b), (a) < (c), (a) < (d), (a) < (e), (b) < (e), (c) < (e)**
**Daily sleep duration**						
Less than 8 hours (a)	45 (19.0)	14.97 ± 5.48	14.29 ± 5.60	14.11 ± 6.05	13.49 ± 6.11	56.87 ± 19.50
8–10 hours (b)	162 (69.0)	12.04 ± 4.44	12.02 ± 4.70	11.56 ± 5.40	11.09 ± 4.90	46.71 ± 15.66
More than 10 hours (c)	28 (12.0)	13.87 ± 5.54	14.55 ± 6.18	13.09 ± 7.09	13.16 ± 7.08	54.68 ± 21.43
***F/p		7.328/0.001**	5.508/0.005*	3.778/0.024*	4.418/0.013*	7.477/0.001**
		**(a) < (b)**	**(a) < (b), (b) < (c)**	**(a) < (b)**	**(a) < (b)**	**(a) < (b), (a) < (c)**
**Recent occurrence of eye pain or itching**						
Yes	29 (12.5)	14.74 ± 5.26	14.85 ± 6.21	13.30 ± 6.54	13.73 ± 6.72	56.62 ± 19.94
Rarely	88 (37.5)	12.98 ± 4.87	13.19 ± 4.82	12.30 ± 5.55	12.04 ± 5.28	50.51 ± 16.55
No	117 (50.0)	12.21 ± 4.77	11.91 ± 4.99	11.92 ± 5.85	11.12 ± 5.28	47.15 ± 17.50
***F/p		3.218/0.042*	4.421/0.013*	0.668/0.514	2.809/0.062	3.621/0.028*
		**(a) < (c)**	**(a) < (c)**			**(a) < (c)**
**Classroom**						
5	84 (36.0)	12.71 ± 4.95	12.84 ± 5.29	12.67 ± 6.08	11.85 ± 5.68	50.07 ± 18.83
6	67 (28.5)	12.48 ± 4.95	12.53 ± 4.89	12.11 ± 5.60	11.85 ± 5.77	48.96 ± 17.02
7	62 (26.5)	13.62 ± 4.90	13.25 ± 5.37	12.19 ± 5.67	11.78 ± 5.38	50.83 ± 17.09
8	21 (9.0)	11.92 ± 4.72	11.68 ± 5.08	11.01 ± 6.10	11.43 ± 4.77	46.04 ± 17.27
***F/p		0.905/0.439	0.538/0.657	0.473/0.701	0.035/0.991	0.430/0.732

*Note: Analyses were conducted using weighted cases, **T-test, ***One-Way ANOVA, *p < .05, **p < .001.

Although income level and parental education did not significantly affect addiction scores (*p* > .05), students from lower and middle-income families exhibited relatively higher average scores than those from higher-income families. A strong association was found between sleep duration and technology addiction. Students who slept less than 8 hours or more than 10 hours per day had significantly higher addiction scores across all dimensions compared to those with regular sleep durations (8–10 hours) (*p* < .01). In terms of eye health, it was found that students who wore glasses or were diagnosed with a visual problem during screening (case group) had significantly higher total technology addiction scores compared to the control group (*p* < .05). Finally, recent experience of eye discomfort (pain or itching) was significantly associated with higher technology addiction scores, especially in the total score, social media, and instant messaging subdimensions (*p* < .05), indicating a potential link between screen overexposure and visual strain (Table 2).

In this study, a Pearson correlation analysis was conducted to examine the relationships between technology addiction and various demographic and behavioural variables. Participants’ average time spent on desktop computers (*r* = .274; *p* < .001), laptops (*r* = .281; *p* < .001), smartphones (*r* = .366; *p* < .001), and online games (*r* = .409; *p* < .001) was positively and significantly correlated with technology addiction scores. These findings suggest that increased screen time across various digital devices is associated with higher levels of technology addiction. Conversely, a significant negative correlation was found between daily sleep duration and total technology addiction scores (*r* = −.111; *p* = .007), indicating that individuals who sleep less tend to report higher levels of digital dependency. Similarly, experiencing eye discomfort such as pain or itching – was negatively correlated with technology addiction (*r* = −.177; *p* < .001), suggesting that extended screen exposure may adversely affect visual health (Table 3).

**Table 3.** Correlation analysis between the studied variables (*n* = 587) Table 3 long description.

Variable		(1)	(2)	(3)	(4)	(5)	(6)	(7)	(8)	(9)	(10)	(11)	(12)	(13)
**Gender (1)**	**r**													
	**p**													
**Income status (2)**	**r**	.069	1											
	**p**	.096	–											
**Average time spent on desktop computer (3)**	**r**	.162**	.050	1										
	**p**	.000	.225	–										
**Average time spent on laptop (4)**	**r**	.085*	.045	.223**	1									
	**p**	.039	.274	.000	–									
**Average time spent on smartphone (5)**	r	−0.10**	0.055	0.244**	0.231**	1								
	**p**	0.813	0.180	**0.000**	**0.000**	–								
**Average time spent in online games (6)**	**r**	.271**	−.022	.456**	.379**	.369**	1							
	**p**	.000	.596	.000	.000	.000	–							
**Daily sleep duration (7)**	**r**	−.046	.041	−.024	−.009	−.040	−.021	1						
	**p**	.268	.322	.558	.822	.337	.612	–						
**Recent occurrence of eye pain or itching (8)**	**r**	.176**	.130**	−.027	−.056	−.142**	−.043	−.028	1					
	**p**	.000	.002	.509	.179	.001	.295	.496	–					
**Technology addiction scale total score (9)**	**r**	.025	−.103*	.274**	.281**	.366**	.409**	−.111**	−.177**	1				
	**p**	.542	.013	.000	.000	.000	.000	.007	.000	–				
**Social Media addiction (10)**	**r**	−.030	−.084*	.181**	.234**	.295**	.323**	−.137**	−.148**	.834**	1			
	**p**	.472	.043	.000	.000	.000	.000	.001	.000	.000	–			
**Instant Messaging addiction (11)**	**r**	−.091*	−.111**	.184**	.191**	.293**	.227**	−.047	−.179**	.802**	.588**	1		
	**p**	.028	.007	.000	.000	.000	.000	.255	.000	.000	.000	–		
**Online Gaming addiction (12)**	**r**	.272**	−.064	.325**	.274**	.292**	.505**	−.090*	−.087*	.786**	.573**	.424**	1	
	**p**	.000	.124	.000	.000	.000	.000	.029	.034	.000	.000	.000		
**Website addiction (13)**	**r**	−.097*	−.080	.192**	.211**	.311**	.259**	−.089*	−.169**	.839**	.586**	.624**	.523**	1
	**p**	.019	.054	.000	.000	.000	.000	.031	.000	.000	.000	.000	.000	–

*Correlation is significant at the 0.05 level (2-tailed). **Correlation is significant at the 0.01 level (2-tailed).

Strong and statistically significant positive correlations were also observed between the total technology addiction score and its subdimensions: social media addiction (*r* = .834), instant messaging addiction (*r* = .802), online gaming addiction (*r* = .786), and website addiction (*r* = .839) (all *p* < .001). These subdimensions also demonstrated high intercorrelations, reinforcing the multidimensional nature of technology addiction. From a demographic perspective, gender was significantly associated with online gaming addiction (*r* = .272; *p* < .001), with male students tending to score higher in this subdomain. Additionally, income level showed a weak but statistically significant negative correlation with technology addiction (*r* = −.103; *p* = .013), indicating that students from lower-income families may be more prone to excessive technology use. From a demographic perspective, gender was significantly associated with online gaming addiction (*r* = .272; *p* < .001), with male students tending to score higher in this subdomain. Additionally, income level showed a weak but statistically significant negative correlation with technology addiction (*r* = −.103; *p* = .013), indicating that students from lower-income families may be more prone to excessive technology use (Table 3).

The logistic regression analysis revealed that the model significantly predicted group membership (case/control) and demonstrated a good fit to the data (Nagelkerke *R* ^2^ = .115; Hosmer–Lemeshow test, *p* = .782). According to the analysis, gender was identified as a significant predictor, with girls being more likely to be in the case group compared to boys (OR = 2.13, 95% CI [1.057, 4.292], *p* = .034). Additionally, an increase in the average daily time spent playing online games was associated with higher odds of being in the case group (OR = 1.665, 95% CI [1.219, 2.276], *p* = .001). Moreover, each one-point increase in the total score of the Technology Addiction Scale was associated with a 2.3% increase in the odds of belonging to the case group (OR = 1.023, 95% CI [1.004, 1.043], *p* = .016). No other variables included in the model significantly predicted group membership (Table 4).

**Table 4.** Predictors of case group membership: results from logistic regression (*n* = 235*) Table 4 long description.

*Predictor variable*	*B*	*S.E.*	*Wald*	*df*	*p-Value*	*Exp(B)*	*95% CI for Exp(B)*
*Gender (1 = female, 2 = male)*	0.756	0.357	4.473	1	.034	2.130	[1.057, 4.292]
*Family income level*	−0.037	0.298	0.015	1	.902	0.964	[0.538, 1.729]
*Avg. daily time spent on mobile phone*	−0.154	0.155	0.996	1	.318	0.857	[0.633, 1.160]
*Avg. daily time spent playing online games*	0.510	0.159	10.253	1	.001	1.665	[1.219, 2.276]
*Eye pain or itching in recent days*	−0.028	0.228	0.015	1	.903	0.973	[0.622, 1.521]
*Total Technology Addiction Scale (TTA) score*	0.023	0.010	5.856	1	.016	1.023	[1.004, 1.043]
*Constant*	−4.296	1.062	16.356	1	.000	0.014	–

Model fit indices: −2 Log Likelihood = 275.569, Nagelkerke *R* ^2^ = .115, Hosmer-Lemeshow Test: *χ* ^2^(8) = 4,766, *p* = .782, *p* < .01–.001, SE, standard error; *β* = standardized regression coefficient; CI, confidence interval.

*Note: Analyses were conducted using weighted cases.

## Discussion

The findings of this study indicate that while overall technology addiction levels did not significantly differ between genders, specific patterns of digital media use did vary. Male students reported higher online gaming addiction, while no gender differences were found in other domains. This aligns with prior research showing boys’ preference for competitive and action-based content, whereas girls engage more in social interaction online (Reid Chassiakos *et al.*, 2016; Livingstone and Blum-Ross, 2020). These results suggest that although the total exposure to digital media may be similar across genders, the type of content preferred and the intended use of technology – such as entertainment versus communication – may differ, reflecting gender-based tendencies in digital engagement. Indeed, a meta-analysis indicated that excessive use of mobile devices particularly increases the risk of myopia, with this effect varying depending on the type of screen, usage time, and the distance to the screen (Ha *et al.*, 2025). Therefore, the gender differences observed in this study are not only indicative of variations in addiction levels but also highlight the need to consider potential health risks associated with different digital habits. This underscores the importance of gender-sensitive digital health education and intervention programmes that address these specific behaviours and associated risks. From a primary health care perspective, these findings are important because technology addiction and excessive screen exposure are modifiable behavioural risk factors. School nurses, primary care professionals, and community health nurses can play a key role in identifying students at risk, monitoring screen-related visual complaints, and providing counselling on digital hygiene, sleep routines, and healthy screen habits. Integrating technology-use assessment into routine school health screenings may therefore strengthen early prevention strategies.

Although prior studies have indicated that parental education levels may influence children’s digital media use and technology addiction risk, the findings of this study did not support a statistically significant association between parents’ educational background and students’ technology addiction scores. This suggests that in this sample, factors other than parental education – such as device usage duration and type of content-may play a more prominent role in the development of technology addiction. Nonetheless, the literature emphasizes the importance of parental involvement and digital literacy. For example, a study has noted that parents with lower educational levels struggle to limit their children’s digital media usage, which increases the risk of addiction (Domoff *et al*., 2019). Lauricella *et al*. (2015) demonstrated that parents with higher education levels are more conscientious in controlling media content and screen time. Although not directly reflected in the statistical outcomes of this study, such evidence highlights the potential protective role of digitally literate and engaged parenting. Therefore, promoting parental digital literacy remains a relevant strategy for mitigating technology-related risks among children and adolescents, especially in increasingly digital post-pandemic environments (Nagata *et al*., 2022). Therefore, this study suggests that improving not only children’s but also parents’ digital literacy could be a crucial strategy in preventing technology addiction. In line with this, it is recommended to expand informational and awareness programs aimed at parents.

Contrary to prior findings, this study did not reveal a significant association between family income and technology addiction. However, children from lower- and middle-income families exhibited higher average scores, suggesting a potential trend toward greater screen dependency in socioeconomically disadvantaged groups, possibly due to limited parental supervision and fewer alternative activities. For example, George *et al.* (2018) and Rideout and Robb (2019) conducted studies indicating that children from low socioeconomic backgrounds spend more time in front of screens and have more limited access to parental supervision and alternative entertainment options. One possible explanation is that digital device ownership and media exposure have become more widespread across all income levels, reducing disparities in access. However, literature continues to emphasize that socio-economic constraints may still influence the quality and context of digital media use. For instance, children in low-income families may rely more heavily on digital devices for entertainment due to fewer structured alternatives (Rideout and Robb, 2019). This situation can reinforce addictive behaviours. Similarly, Twenge and Campbell (2018) emphasized that socioeconomic differences in digital media usage are prominent, with children from low-income families consuming more digital content. Children from socioeconomically disadvantaged backgrounds are more likely to engage in passive and non-educational media use, increasing the risk of technology addiction. Limited opportunities for outdoor activities and social interaction further exacerbate their screen dependency. Therefore, prevention programs must address these economic disparities by promoting digital literacy, strengthening family support, and ensuring access to alternative recreational activities.

The findings related to sleep duration in this study indicate a statistically significant relationship between sleep patterns and technology addiction. Specifically, students who reported sleeping less than 8 hours exhibited significantly higher addiction scores compared to those with more regular sleep durations. This finding aligns with prior study by Paulich *et al.* (2021), which highlighted the inverse relationship between screen time and sleep quality, as well as research by Livingstone and Blum-Ross (2020), which addressed the effects of digital devices on children’s daily routines. The increased use of digital devices, particularly late at night, may interfere with circadian rhythms, contributing to insufficient or irregular sleep. These findings emphasize the importance of healthy digital habits to prevent technology-related sleep problems among adolescents Chang and Lee (2024).

In addition, the present study found a strong, statistically significant positive correlation between increased screen time – especially on devices such as laptops, smartphones, and for online gaming – and higher technology addiction scores (*p* < .01). As students’ daily screen exposure increased, their addiction scores across all subdimensions (social media, instant messaging, online gaming, and web browsing) also rose notably. These findings are consistent with previous research, which identified the harmful effects of excessive screen time on children’s cognitive, social, and emotional development (Reid Chassiakos *et al.*, 2016). Similarly, it aligns with the work of Priftis and Panagiotakos (2023) emphasized that high levels of technology dependence can adversely affect both the mental and physical health of children. Taken together, the data underscore the urgent need for strategies aimed at regulating screen use and promoting balanced digital behaviours among school-aged youth.

The increasing use of visual-auditory digital media among children has been associated not only with technology addiction but also with health issues such as myopia and digital eye strain (Foreman *et al.*, 2021; Ha *et al.*, 2025). In line with this, one of the notable findings of this study is the relationship between students’ recent eye problems – such as pain or itching – and elevated technology addiction scores. Students who reported experiencing recent eye discomfort had significantly higher scores in the total technology addiction scale, as well as in the social media and instant messaging subdimensions. These results suggest that prolonged screen exposure, particularly at close distances, may place considerable strain on children’s visual systems, reinforcing the risk of digital eye strain (Almutairi *et al*., 2024). Recently, students experiencing eye problems had significantly higher technology addiction scores, supporting the notion that prolonged use of digital devices has a detrimental effect on visual health.

The present findings also support the association between technology addiction and visual complaints among early adolescents. Students who reported recent eye discomfort, such as pain or itching, had higher total technology addiction scores, particularly in the social media and instant messaging subdimensions. This finding is consistent with previous evidence linking prolonged screen exposure with digital eye strain and myopia (Foreman *et al.*, 2021; Jain *et al.*, 2023; Ha *et al.*, 2025). Excessive close-distance screen use may increase visual load and contribute to symptoms such as eye discomfort, blurred vision, and difficulty focusing. Therefore, the higher addiction scores observed among students with visual problems should be interpreted as an important behavioural risk indicator. These results emphasize the need for early screening, digital hygiene education, and guidance on balanced screen use in school and primary health care settings.

## Limitations

While this study provides valuable insights into the relationship between technology addiction and various factors such as gender, parental education, and socioeconomic status, several limitations should be acknowledged. The sample may not fully represent the broader adolescent population, limiting generalizability. The reliance on self-reported data introduces potential bias due to over- or underreporting. Moreover, the cross-sectional design prevents causal inference between technology addiction and associated factors.

## Conclusion

This multicentre case–control study investigated whether technology addiction predicts visual problems in early adolescents. The findings indicate that students with higher technology addiction scores – particularly in online gaming, social media use, and instant messaging – are at greater risk for visual issues, highlighting technology overuse as a modifiable behavioural predictor rather than a direct cause. Consistent with previous research, prolonged screen exposure and irregular sleep patterns were significantly associated with elevated addiction levels and ocular complaints. Gender differences in technology use behaviours were evident: boys exhibited higher gaming addiction scores, whereas girls scored higher in instant messaging and website use. These patterns suggest that targeted preventive strategies may need to consider gender-specific digital habits. Socioeconomic factors did not directly predict visual problems in this sample, suggesting that behavioural patterns of technology use may outweigh demographic characteristics as predictors within early adolescence. However, environmental and family context may still indirectly influence screen behaviours, which warrants further investigation in future studies.

From a primary health care perspective, these findings underscore the importance of early risk identification and behavioural assessment. School and community health nurses are ideally positioned to screen for high-risk digital behaviours, monitor associated sleep and vision complaints, and provide tailored counselling to both students and caregivers. Integrating such assessments into routine school health check-ups or primary care visits could enable timely interventions and preventive guidance, ultimately supporting healthier digital habits and reducing the likelihood of visual problems. Moreover, recognizing technology addiction as a predictive behavioural factor allows health professionals to prioritize preventive interventions and digital literacy education, rather than solely focusing on reactive treatment of visual complaints. Future research should explore longitudinal designs to confirm predictive pathways and evaluate the effectiveness of nurse-led interventions in reducing technology-related visual problems.

## Data Availability

The data are available upon reasonable request from the authors.

## Table 1. Long description

The table compares demographic characteristics of case-control groups. It has 20 rows and 8 columns. The columns are Characteristics, Overall (n = 587), Control (n = 540), Case (n = 47), F, X squared, and p-Value. The rows include various demographic factors such as gender, classroom, mother’s education level, father’s education level, income status, owning a smartphone, owning a desktop computer, owning a laptop, average time spent on cable computer, average time spent on laptop, average time spent on smartphone, average time spent on online games, daily sleep duration, recent occurrence of eye pain or itching, and prevalence of technology addiction. Each row provides the count and percentage for each characteristic in the overall, control, and case groups, along with statistical values for F, X squared, and p-Value.

Navigate back to Table 1..

## Table 2. Long description

The table presents an analysis of demographic variables related to technology addiction, with 235 participants. It includes columns for internal code addition, mean, standard deviation, technology addiction subscale, and p-value. The table is divided into sections such as case and control groups, gender, educational stage, and device usage. Each row lists specific variables and their corresponding statistical values. Notable trends include higher mean scores in the case group across all subdimensions of technology addiction compared to the control group. Boys show significantly higher scores in online gaming addiction, while older students report higher addiction levels in online gaming. Higher screen time is consistently associated with increased technology addiction scores.

Navigate back to Table 2..

## Table 3. Long description

A table with 13 rows and 13 columns showing correlation analysis between various variables. The table includes correlation coefficients (r) and p-values (p) for each pair of variables. The variables studied are gender, income status, average time spent on desktop computer, average time spent on laptop, average time spent on smartphone, average time spent in online games, daily sleep duration, recent occurrence of eye pain or itching, technology addiction scale total score, social media addiction, instant messaging addiction, online gaming addiction, and website addiction. Each cell in the table contains a correlation coefficient and a p-value, indicating the strength and significance of the relationship between the variables. Notable trends include positive correlations between technology addiction and time spent on various devices, and negative correlations between technology addiction and daily sleep duration.

Navigate back to Table 3..

## Table 4. Long description

A table titled Predictors of case group membership: results from logistic regression (n = 235) with eight rows and nine columns. The columns are labeled Predictor variable, B, S.E., Wald, df, p-Value, Exp(B), and 95% CI for Exp(B). The rows are labeled with different predictor variables: Gender, Family income level, Avg. daily time spent on mobile phone, Avg. daily time spent playing online games, Eye pain or itching in recent days, Total Technology Addiction Scale (TTA) score, and Constant. Each row contains values for each column, representing the results of the logistic regression analysis. Notable values include significant p-values for Gender (p = .034), Avg. daily time spent playing online games (p = .001), and Total Technology Addiction Scale (TTA) score (p = .016).

Navigate back to Table 4..
